# Development of an openEHR Template for COVID-19 Based on Clinical Guidelines

**DOI:** 10.2196/20239

**Published:** 2020-06-10

**Authors:** Mengyang Li, Heather Leslie, Bin Qi, Shan Nan, Hongshuo Feng, Hailing Cai, Xudong Lu, Huilong Duan

**Affiliations:** 1 College of Biomedical Engineering and Instrument Science Zhejiang University Hangzhou, Zhejiang China; 2 Key Laboratory for Biomedical Engineering Ministry of Education Hangzhou, Zhejiang China; 3 Atomica Informatics Melbourne Australia; 4 openEHR Foundation London United Kingdom; 5 Hangzhou Joyrun Medical Technology Cooperation Hangzhou, Zhejiang China; 6 School of Industrial Engineering Eindhoven University of Technology Eindhoven Netherlands

**Keywords:** coronavirus disease, COVID-19, openEHR, archetype, template, knowledge modeling, clinical guidelines

## Abstract

**Background:**

The coronavirus disease (COVID-19) was discovered in China in December 2019. It has developed into a threatening international public health emergency. With the exception of China, the number of cases continues to increase worldwide. A number of studies about disease diagnosis and treatment have been carried out, and many clinically proven effective results have been achieved. Although information technology can improve the transferring of such knowledge to clinical practice rapidly, data interoperability is still a challenge due to the heterogeneous nature of hospital information systems. This issue becomes even more serious if the knowledge for diagnosis and treatment is updated rapidly as is the case for COVID-19. An open, semantic-sharing, and collaborative-information modeling framework is needed to rapidly develop a shared data model for exchanging data among systems. openEHR is such a framework and is supported by many open software packages that help to promote information sharing and interoperability.

**Objective:**

This study aims to develop a shared data model based on the openEHR modeling approach to improve the interoperability among systems for the diagnosis and treatment of COVID-19.

**Methods:**

The latest Guideline of COVID-19 Diagnosis and Treatment in China was selected as the knowledge source for modeling. First, the guideline was analyzed and the data items used for diagnosis and treatment, and management were extracted. Second, the data items were classified and further organized into domain concepts with a mind map. Third, searching was executed in the international openEHR Clinical Knowledge Manager (CKM) to find the existing archetypes that could represent the concepts. New archetypes were developed for those concepts that could not be found. Fourth, these archetypes were further organized into a template using Ocean Template Editor. Fifth, a test case of data exchanging between the clinical data repository and clinical decision support system based on the template was conducted to verify the feasibility of the study.

**Results:**

A total of 203 data items were extracted from the guideline in China, and 16 domain concepts (16 leaf nodes in the mind map) were organized. There were 22 archetypes used to develop the template for all data items extracted from the guideline. All of them could be found in the CKM and reused directly. The archetypes and templates were reviewed and finally released in a public project within the CKM. The test case showed that the template can facilitate the data exchange and meet the requirements of decision support.

**Conclusions:**

This study has developed the openEHR template for COVID-19 based on the latest guideline from China using openEHR modeling methodology. It represented the capability of the methodology for rapidly modeling and sharing knowledge through reusing the existing archetypes, which is especially useful in a new and fast-changing area such as with COVID-19.

## Introduction

The coronavirus disease (COVID-19) is a severe infectious disease that has been confirmed to lead to human-to-human transmission since December 2019 [1]. Considering the sudden outbreak of the disease as a challenging threat, it was brought into the Class B of infectious diseases defined in the Law of the People’s Republic of China on the Prevention and Control of Infectious Diseases, but management and policies were adopted according to the Class A of infectious diseases [2,3]. All of the challenges have brought great pressure on medical institutions and professionals, including the lack of medical equipment and the complexity of diagnosis. At the same time, the fear of the disease has a negative impact on both the psychological and physiological well-being of affected individuals.

Although, most cases of the disease occurred in mainland China in the beginning, other areas have also confirmed cases of the same disease, and the number of cases continues to increase. The World Health Organization (WHO) has declared the 2019-20 coronavirus outbreak to be a Public Health Emergency of International Concern [4]. So far, the United States is the most seriously affected country in the world. In addition to China, many cases were found in the Western Pacific Region, such as South Korea and Japan. Russia and Great Britain were relatively seriously affected areas in Europe [5] according to the latest data reported by the WHO.

Symptoms include fever, cough, or shortness of breath, and even pneumonia, multi-organ failure, and death in the most severe cases. The latent period can be between 1 and 14 days, and on average is between 3 and 7 days according to the epidemiological investigation. What is even worse is that some patients may be asymptomatic at the beginning, which results in some undetected errors [6]. Given the severity of the infectious disease and urgency of diagnosis and treatment, a large number of studies related to disease prevention and control have been carried out with the support of various countries according to the WHO [7].

Considering the rapid spread of the disease, the transferring of knowledge of diagnosis and treatment of the disease and newly updated achievements of research are important, especially from areas with improved epidemics like mainland China, the area where the epidemic had begun. Although some efforts have been made through teleconsultation and medical staff assistance, they are still limited due to a lack of experts. Using decision support tools is an efficient way to transfer the knowledge of experts to clinical practices. Epic (Epic Systems), which is a health care software company with electronic medical record software application, has sent out an update to its customers to detect potential cases of COVID-19 [8]. DIPS, which is a major openEHR vendor, released open source components to assist software developers creating apps to fight COVID-19 [9]. Although Epic provided a complete solution for medical information systems where the clinical decision support tools have already been embedded, most other systems still need to integrate with medical information systems to be used in clinical practice. The interoperability of disease-related data has become an important issue.

Many studies on terminology standardization have been conducted to improve the interoperability. Systematized Nomenclature of Human Medicine International has issued an interim release to promote the analysis with the most up-to-date terminology [10]. The Observational Health Data Sciences and Informatics has also committed vocabulary about COVID-19 into GitHub [11]. These efforts have been focused on the shared representation of the concepts related to COVID-19 but are not enough for data exchanging, with the shared data model being the key issue. It specifies not only the data structures but also the attributes of data elements. Although there exist many methods [12-15] to develop a data model for certain requirements, the rapidly updating knowledge for COVID-19 makes it a challenge to achieve a flexible model constantly with the requirement evolution.

An open, semantic-sharing, and collaborative-modeling framework is needed to meet the dynamic change of data requirements. openEHR specifications can be used to create standards and build information and interoperability solutions for health care as a multilevel modeling framework [16]. In the approach suggested by openEHR, the reference model (RM) focuses on the logic structures and attributes required to express data, so it is stable and provides basic components for building concrete medical information models. The archetype model is comprised of archetypes and templates. Based on the RM, archetypes can be developed to define all the attributes about specific clinical concepts. Different archetypes can be organized into context-specific data sets, templates that are mostly developed and used locally. Only the RM is implemented in apps, while clinical information (archetypes and templates) is independent of specific implementations. The approach allows data models to be flexible and extensible within the constraints of the RM, which can keep up with the development of the clinical knowledge and meet the requirements of the complicated clinical environment [17]. The knowledge obtained in clinical practice in mainland China can be sharable and beneficial for other countries and regions by being formalized as openEHR archetypes and templates.

As a goal, we developed an openEHR template to promote interoperability among clinical systems for the diagnosis and treatment of COVID-19. The remainder of this paper is organized as follows: the Methods section introduces the knowledge source and methodology we used to develop and review the template, the Results section illustrates our results step-by-step according to the proposed methodology, and the Discussion section discusses the contributions of this paper and limitations.

## Methods

### Knowledge Sources

Given that the outbreak of the disease happened within a short time frame, the involved knowledge is limited. To make them justified and believed, the Guideline for Diagnosis and Treatment of COVID-19 released by the National Health Commission of the People’s Republic of China was adopted as the knowledge source. At present, the guideline has evolved to the 7th edition [18] (the English translated version is also available [19]). The guideline can be divided into 13 sections: (1) Pathogenic Characteristics; (2) Epidemiological Characteristics; (3) Pathological Changes; (4) Clinical Characteristics; (5) Diagnostic Criteria; (6) Clinical Classifications; (7) Clinical Warning Signs for Severe and Critical Cases; (8) Differential Diagnosis; (9) Discovery and Reporting of Cases; (10) Treatment; (11) Criteria for Discharge and Notes After Discharge; (12) Transportation Principles; and (13) Prevention of Infection in Medical Establishments. Since only the contents related to conditions and recommendations for diagnosis and treatment will be used for data exchanging, sections 4-9, section 11, and part of section 10 were selected as the knowledge source. There are three reasons for excluding other sections. First, sections 1-3 describe the general knowledge of COVID-19, most of which will not be directly used as the condition for the judgment of diagnosis, treatment, and management. Second, sections 12 and 13 illustrate the regulations and policies for transportation and infection prevention, and will also not be used for diagnosis and treatment. Third, in another part of section 10, traditional Chinese medicine and herbal medicine are regarded as a kind of alternative medicine, which is a supplement to evidence-based medicine and has only regional characteristics.

### The Process of Development

**Figure 1:**
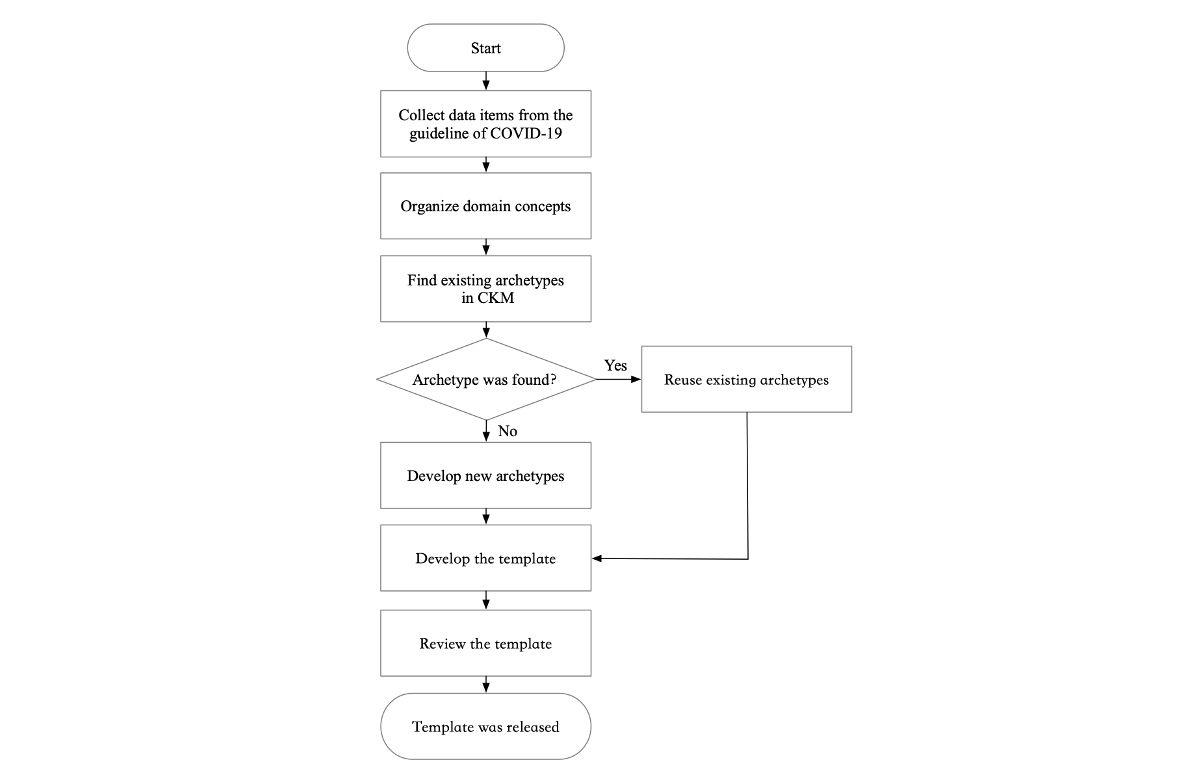
The method of developing an openEHR template about COVID-19. CKM: Clinical Knowledge Manager; COVID-19: coronavirus disease.

To develop an openEHR template for COVID-19, our method consists of six steps. These steps include collecting data items, organizing domain concepts, searching corresponding archetypes, developing an openEHR template, and reviewing and releasing the template (see Figure 1).

#### Collecting Data Items

In this step, data items related to diagnosis and treatment were extracted from sections 4-9, section 11, and part of section 10 of the guideline, and further organized in Excel (Microsoft Corporation) with three columns. The first and the second column corresponded to the sections and subsections of the guideline, and the third column corresponded to the data items extracted from the subsection. The extracted original data items in Chinese have been translated from Chinese to English. Although extraction of data items was done manually, two principles were followed to lower the bias of extraction and reduce the errors.

First, considering that the composition of the guideline is hierarchical and segmented, which is the inherent knowledge of grouping data items, the extracted data items were organized in the same hierarchical structure as the guideline to not only lay the foundation for further organization of domain concepts but also provide a much easier correspondence for reviewers when verifying the correctness of the extraction.

Second, two members of our team extracted these data items separately. After the extraction, both of them exchanged and reviewed the opponent’s results. For the results acknowledged by both of them, they were included in the Excel file directly. For the results acknowledged by only one of them, they were reviewed by another member to confirm the final results. For the results that were acknowledged by both of them but needed to be refined, they were re-extracted by both team members.

#### Organization of Domain Concepts

The organization of domain concepts is the basis for the development of archetypes and templates. Five steps were performed to organize domain concepts from the extracted data items.

1. If the data items from different subsections were the same semantically, they were merged into a single-data item. For example, coagulopathy and blood coagulation disorder can be merged into blood coagulation disorder.
2. If the data items from different subsections belong to the same domain concept, they were regrouped into a more suitable group other than the sections or subsections. For example, symptoms such as fever and difficulties in breathing found in different sections will be regrouped together.
3. According to practices from clinical decision support with the clinician participants, medical concepts that are encountered and used commonly were selected and organized as a supplement. For example, in diagnosis and treatment, operations such as surgery are generally mentioned, such as pneumonectomy or splenectomy, but similar medical concepts are not mentioned in the guideline, so this step is a significant supplement to the knowledge extracted from the guideline.
4. All domain concepts were then organized as a tree structure according to the inherent correlation among them and were represented into a mind map using XMind (XMind Ltd) as a tool. The extracted data items can be either the data elements themselves or one value in the value set of the data elements within domain concepts. For example, respiratory failure and blood coagulation disorder are each treated as a single value within the value set of diagnoses.
5. Finally, the domain concepts were further classified into three categories according to the different stages in the process of clinical diagnosis and treatment; they are “Instruction, Evaluation, and Observation.”

#### Searching in Clinical Knowledge Manager and Archetype Development

To avoid developing archetypes repeatedly and to facilitate semantic interoperability, the adoption of existing archetypes is of much significance. The openEHR Foundation provides a website called Clinical Knowledge Manager (CKM) [20], which supports international domain knowledge governance and collaborative development of clinical knowledge resources beyond a library of openEHR archetypes and templates.

This step mainly focused on performing a search in the repository to find the archetypes with similar semantics. The name of domain concepts and data items were used as keywords to identify the archetypes. On account of polysemy and synonym, extra manual work was carried out to find the related archetypes. Some archetypes can be used directly, which means the data elements can be represented in these archetypes exactly, and there is no difference on a semantic level among them.

If no corresponding archetypes exist or existing archetypes cannot represent the data elements fully, developing new archetypes or extending existing archetypes is necessary according to the syntax of openEHR Archetype Definition Language [21].

#### Development of openEHR Template

After the required archetypes were found and developed, the template can be built based on them. This task can be performed with the support of Ocean Template Editor [22] to set constraints on these archetypes to fit the requirements for data exchanging. In this step, two issues come in to focus. First, constraints about terminology for certain data elements in the archetype should be made, such as medication. For example, the name of drugs can be generic names that can be identified around the world and aliases, which can be used in specific countries or regions. A unified value domain can be significant for data sharing and interoperability. Second, from the perspective of diagnosis and treatment, time series problems should be taken into consideration. For example, “patients whose chest imaging shows a significant progression of lesions (>50%) are managed as a severe case within 24-48 hours” was mentioned in the guideline. To support the diagnosis decision, the occurrence of the data elements in the template should be more than one so that they can contain the required two elements including the first chest imaging and the second chest imaging within 24-48 hours.

#### Review and Release of the Template

The review process is necessary to achieve a template with high quality. Two aspects of the template have been reviewed. First, the representation of the domain knowledge, such as correctness of the semantics, classification of data elements, and logic structure of archetypes, was reviewed. Second, the template was reviewed from informatics, such as the data types of data elements and relationships among different archetypes. The study has designed two review phases to achieve the goal.

1. Internal review phase: the internal review group includes a total of four persons, with one person that is familiar with the COVID-19 guideline, two persons who are developers of clinical data repositories (CDRs) and decision support tools, and one person that is familiar with openEHR specifications.
2. Outreach review phase: the participation of two domain experts beyond the research team was involved in this phase. One has the expertise of openEHR modeling and the other has the expertise of medical informatics.

After the review, the template and the used archetypes have been uploaded and shared in the Healthcare Modeling Collaboration [23] and in the project of the CKM.

### Verification of the openEHR Template COVID-19

The study has designed a test case to verify the feasibility of the template. The case was conducted in a hospital located in Wuhan, which has already implemented openEHR-based CDR and accepted a large number of patients with COVID-19. The CDR in the hospital was built based on the solution we have proposed, which can be found in [24,25]. With a developed openEHR model, the storage structure can be generated easily. The decision support tool for diagnosis and treatment of COVID-19 has been developed and is planned to be used in the practice. There is great demand for data sharing and interoperability between CDR and the decision support tool.

**Figure 2:**
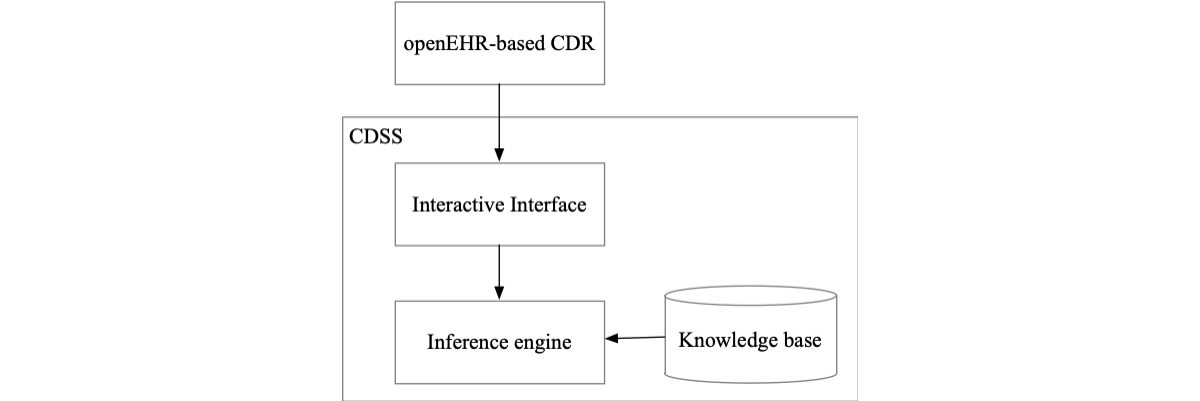
The interaction diagram between CDR and decision support tool. CDR: clinical data repository; CDSS: clinical decision support system.

The test case was designed to include two steps: (1) the template was applied to the openEHR-based CDR, which can provide template-specific data query and storage service through application programming interfaces (APIs); and (2) the decision support tool used these APIs to query the data useful for the judgment of the diagnosis and treatment. The interaction diagram is shown in Figure 2.

## Results

**Figure 3:**
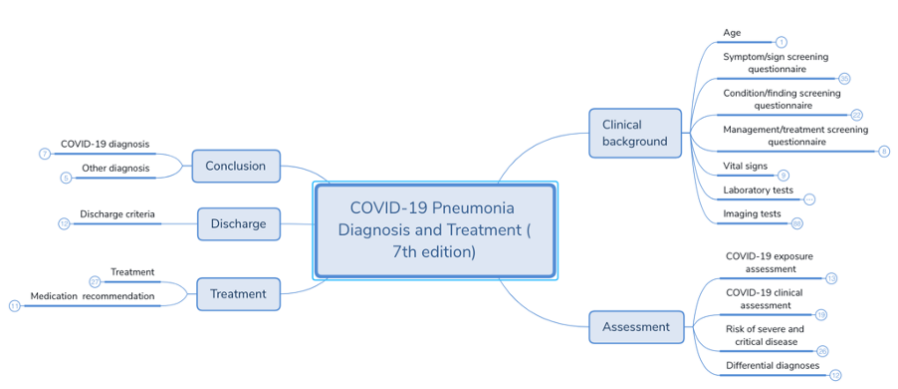
Domain concepts about COVID-19. COVID-19: coronavirus disease.

Based on the methodology previously described, 203 data items were extracted from the guideline in China, including 8 sections and 15 subsections (see Multimedia Appendix 1). After the classification and merge of these data elements, 16 domain concepts (16 leaf nodes in the mind map) were organized for diagnosis and treatment of COVID-19. The results in this step are illustrated in the mind map in Figure 3 (full results can be found in Multimedia Appendix 2).

**Figure 4:**
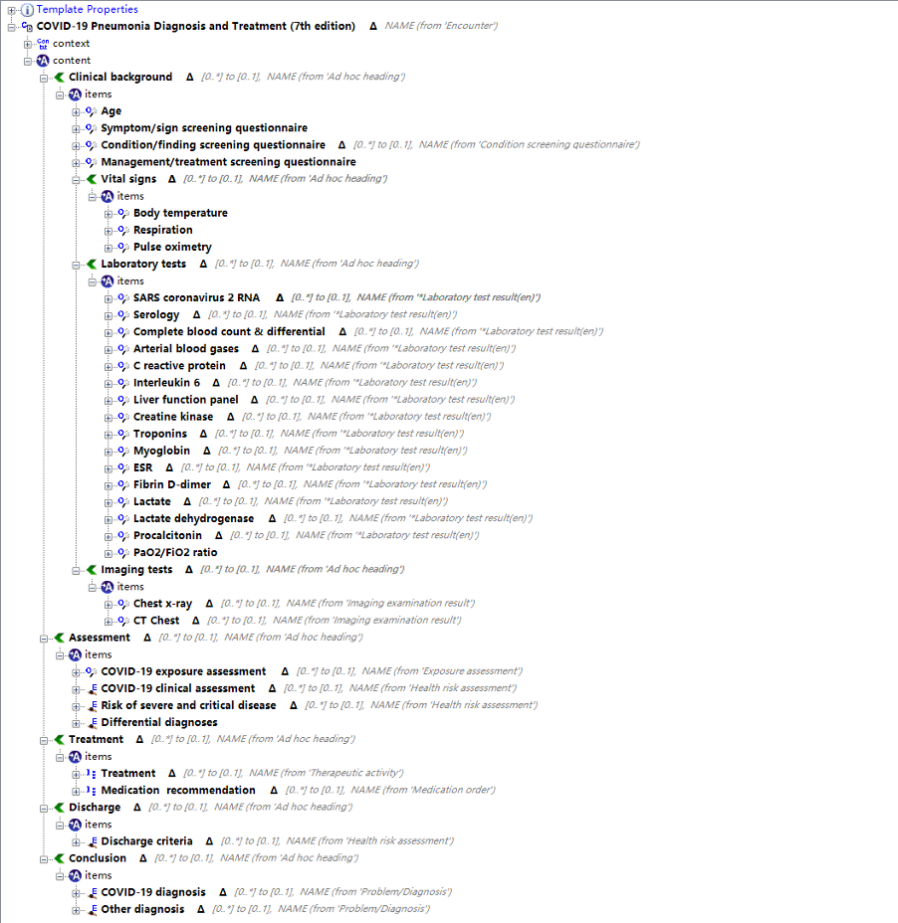
The developed template in Ocean Template Editor. COVID-19: coronavirus disease; CT: computed tomography; ESR: erythrocyte sedimentation rate; SARS: severe acute respiratory syndrome.

Among these domain concepts, only 2 archetypes were classified into Instruction, and 3 of them were classified into Evaluation. The archetypes of Observation include 11 items. A total 22 archetypes have been developed to represent all data elements about COVID-19, and all of them can be referred to from the CKM directly. These archetypes found in the CKM, which are adapted to our requirements, are shown in Textbox 1. Finally, a template was developed with the constraint of these archetypes as shown in Figure 4.

**Figure 5:**
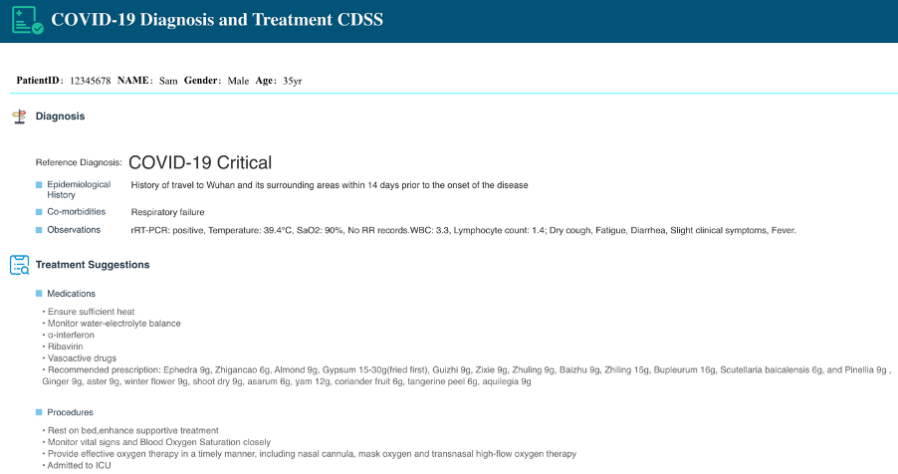
The data view of COVID-19 Diagnosis and Treatment CDSS. CDSS: clinical decision support system; COVID-19: coronavirus disease; ICU: intensive care unit; rRT-PCR: real time reverse transcription-polymerase chain reaction; WBC: white blood cell.

In addition, it has been deployed in a hospital, which has accepted many cases of COVID-19, to support data sharing between CDRs and clinical decision support systems (CDSS). Because the CDR is developed based on openEHR, the storage structure is consistent with the template. Although there exist many storage implementations of openEHR [25,26], they are transparent for the invocation of data services as a result of openEHR two-level modeling. Data elements in the template can be uniquely identified by paths and attributes. In this way, data for each data element can be transferred in representational state transfer (REST)ful API. The rules in CDSS were also built based on the template. The template was used in both CDR and CDSS. The data from RESTful API was parsed, extracted, and used in the inference engine to produce the decision for the case. The user interface for the data view whose data was from openEHR-based CDRs is shown in Figure 5. Textbox 2 shows the part of the content of the data that is in the RESTful API from CDR.

In the end, the template (COVID-19 Pneumonia Diagnosis and Treatment [7th edition]) has been uploaded into the CKM [27].

**Domain concepts and their archetypes found in the Clinical Knowledge Manager.**

**Diagnosis and treatment of the coronavirus disease (COVID-19)**

- openEHR-EHR-COMPOSITION.encounter.v1

**—**

- openEHR-EHR-SECTION.adhoc.v1

**Age**

- openEHR-EHR-OBSERVATION.age.v0

**Symptom and sign screening questionnaire**

- openEHR-EHR-OBSERVATION.symptom_sign_screening.v0

**Condition and findings screening questionnaire**

- openEHR-EHR-OBSERVATION.condition_screening.v0

**Management and treatment screening questionnaire**

- openEHR-EHR-OBSERVATION.management_screening.v0

**Vital signs**

- openEHR-EHR-OBSERVATION.body_temperature.v2
- openEHR-EHR-OBSERVATION.respiration.v2
- openEHR-EHR-OBSERVATION.pulse_oximetry.v1

**Laboratory tests**

- openEHR-EHR-OBSERVATION.laboratory_test_result.v1
- openEHR-EHR-CLUSTER.specimen.v1
- openEHR-EHR-CLUSTER.laboratory_test_analyte.v1
- openEHR-EHR-CLUSTER.inspired_oxygen.v1
- openEHR-EHR-OBSERVATION.pf_ratio.v0

**Imaging tests**

- openEHR-EHR-OBSERVATION.imaging_exam_result.v0
- openEHR-EHR-CLUSTER.imaging_finding.v0

**COVID-19 exposure assessment**

- openEHR-EHR-OBSERVATION.exposure_assessment.v0

**COVID-19 clinical assessment**

- openEHR-EHR-EVALUATION.health_risk.v1

**Risk of severe and critical disease**

- openEHR-EHR-EVALUATION.health_risk.v1

**Differential diagnoses**

- openEHR-EHR-EVALUATION.differential_diagnoses.v0

**Treatment**

- openEHR-EHR-INSTRUCTION.therapeutic_activity_order.v0

**Medication recommendation**

- openEHR-EHR-INSTRUCTION.medication_order.v2

**Discharge criteria**

- openEHR-EHR-EVALUATION.health_risk.v1

**COVID-19 diagnosis**

- openEHR-EHR-EVALUATION.problem_diagnosis.v1

**Other diagnosis**

- openEHR-EHR-EVALUATION.problem_diagnosis.v1

**The part of the content of data in representational state transfer application programming interface from clinical data repositories.**

{

     “labTestResultList”: [

               {

                            “itemName”: “Lymphocyte count”,

                            “result”: 1.4,

                            “status”: “”,

                            “unit”: “”

               },

               {

                            “itemName”: “WBC”,

                            “result”: 3.3,

                            “status”: “”,

                            “unit”: “”

               },

               {

                            “itemName”: “rRT-PCR”,

                            “result”: “positive”,

                            “status”: “”,

                            “unit”: “”

               }

       ],

       “medicalRecordList”: [

               {

                            “dateTime”: “”,

                            “text”: “History of travel to Wuhan and its surrounding areas within 14 days prior to the onset of the disease”,

                            “type”: “Epidemic History”

               }

       ],

       “patientInfo”: {

               “bloodType”: [],

               “dateOfBirth”: “1985-01-01”,

               “name”: “Sam”,

               “patientId”: 12345678,

               “sex”: “Male”

       },

       “physicalSignList”: [

               {

                            “itemCode”: “”,

                            “itemName”: “SpO2”,

                            “measureDateTime”: “”,

                            “unit”: “%”,

                            “value”: “90”

               },

               {

                            “itemCode”: “”,

                            “itemName”: “Body_temperature”,

                            “measureDateTime”: “”,

                            “unit”: “℃”,

                            “value”: “39.4”

               },

               {

                            “itemCode”: “”,

                            “itemName”: “RR”,

                            “measureDateTime”: “”,

                            “unit”: “”,

                            “value”: “”

               }

               ],

       “symptomList”: [

               {

                            “reportDateTime”: “”,

                            “text”: “Dry cough, Fatigue, Diarrhea, Slight clinical symptoms, Fever”

               }

       ]

}

## Discussion

### The Template Facilitates the Interoperability in Different Clinical Scenarios

Since the openEHR template developed in our study covered the contents related to clinical characteristics, diagnosis criteria, clinical classification, warning signs for severe and critical cases, differential diagnosis, diagnosis of suspected cases, treatment, and discharge from the latest guideline, it could be used for data exchanging among systems in different clinical scenarios such as screening patients in outpatient clinics, where the diagnosis of suspected cases will be the main focus; the routine round in the wards, where the diagnosis and warning signs for severe or critical cases will be more important; and the intensive care unit, where the treatment recommendation will be the most necessary. Although some hospital information system vendors [8] provided the monolithic solution, the applications for different scenarios with an integrated solution were still encouraged to fully use the expertise of different vendors, which is the norm in the health care institutions. For this reason, the data interoperability is an important issue, and the results of our study can play a significant role.

Furthermore, the results of our study can be used for purposes other than the diagnosis and treatment of COVID-19. It can help to develop scales according to severity at different levels and be used for risk assessment of COVID-19. Meanwhile, it is also significant for the prevention and control of the disease in the community. The questionnaires can be designed for people who are under closed management to monitor their physical conditions.

### openEHR Modeling Approach Is Flexible for Rapidly Changing Knowledge

COVID-19 was a new threatening infectious disease that brought great pressure on medical systems around the world and with limited previous knowledge in the domain. The methods of diagnosis and treatment have been updated rapidly to reflect the achievement of the latest research since the outbreak of the disease, which sets the challenge for data exchange among systems. The openEHR modeling approach perfectly meets the requirement since the multilevel modeling is especially suitable for the knowledge evolution. In the openEHR ecosystem, when the knowledge of diagnosis and treatment has been updated, only the template needs to be updated and the apps can be kept unchanged. This enables the latest knowledge to be applied to clinical practice at the fastest speed.

**Figure 6:**
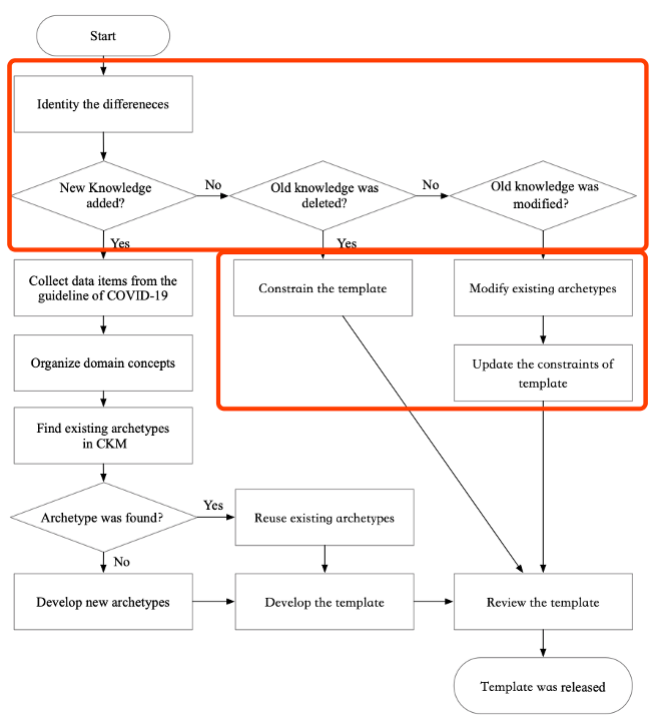
The updating process of the template. CKM: Clinical Knowledge Manager; COVID-19: coronavirus disease.

In our study, once the latest guideline has been released, the new knowledge can be incorporated into the existing template according to the flowchart shown in Figure 6. Compared with starting from scratch as in the first round, only a few steps need to be performed when the knowledge has been updated, which is shown in the red box (Figure 6).

### The Purpose of Modeling Needs to be Refined for Modeling Precisely

The purpose of the template needs to be refined before modeling since it will largely affect the final results. First, although the purpose of the study is to develop the template for diagnosis and treatment, it still needs to be refined based on whether it is for rule-based decision support tools only or general decision support. As an example, the guideline only mentioned that pregnancy status may affect the intervention without specifying the exact rules, so it is not necessary to be modeled if only for the rule-based decision support tool, but it will still be useful information for professionals to make the decision. Second, the refined purpose should also clarify whether it is used for exchanging the original data from EMR or the condition points for the final decision support. Data items extracted from the guideline usually did not exist in CDR, so they need to be abstracted from the existing data. For instance, the guideline may describe the “Two consecutive negative nucleic acid tests using respiratory tract samples (taken at least 24 hours apart)” as a condition point for discharge, but it has to be calculated from two data items of the nucleic acid test.

### Is it Enough to Use the Latest Guideline as the Only Knowledge Source?

This study has used the guideline released by the National Health Commission of the People’s Republic of China as the only knowledge source for modeling since the authoritative knowledge was limited at the beginning of the outbreak. However, with the ever-increasing research results and experiences of diagnosis and treatment, there are and will be more knowledge sources, such as the handbook developed by the First Affiliated Hospital, Zhejiang University School of Medicine jointly sponsored by the Jack Ma Foundation and Alibaba Foundation [28], which could be taken as complementary to the guideline. Our modeling approach makes it much easier to apply new or updated knowledge in the template quickly.

### More Case Studies and Reviews Aare Needed

Although the template has been reviewed and verified in our study, it still has limitations. First, due to the reason that most of the experienced medical professionals were prioritizing clinical care of patients with COVID 19, it was difficult to have the template reviewed by professionals. Second, since there are not many cases that have been conducted, there may exist some points not appropriate for specific cases (eg, the patient may need to be treated with extra intervention that is not represented in the template). Therefore, further case studies and reviews are necessary to improve the template.

### Limitations of Using openEHR Template in an Actual Hospital

The template can be easily deployed in the openEHR-based CDR as shown in this study. However, not all institution’s implemented systems are based on openEHR, so there will be a limitation for the use of the template in such scenarios. However, since the infrastructure of openEHR has been designed to be compatible with other existing industry standards, the template can be easily transferred to other popular accepted industry standards like JavaScript Object Notation (JSON), XML, and Health Level 7. To take JSON as an example, the template can be expressed in this format within the support of the JSON schema [29]. The JSON schema is similar to the XML schema to help describe the data format and provide the constraints for the data expression.

### Conclusions

This paper developed and released the openEHR template based on the latest guidelines of COVID-19 in China. Most of the archetypes used in the template can be covered by existing archetypes in the CKM. This study proved that the openEHR approach has advantages in modeling a new medical application field and meeting the requirements of rapidly updating knowledge. The template developed in this study could be used to transfer the experience and knowledge achieved from China to other countries and regions as soon as possible from the perspective of improving data exchange among applications to defeat COVID-19.

## Appendix

Multimedia Appendix 1 Data items extracted from the latest clinical guideline released by China.

Multimedia Appendix 2 The details of domain concepts presented in mind map.

## Abbreviations

API: application programming interface
CDR: clinical data repository
CDSS: clinical decision support system
CKM: Clinical Knowledge Manager
COVID-19: coronavirus disease
JSON: JavaScript Object Notation
REST: representational state transfer
RM: reference model
WHO: World Health Organization
