# Supplementary figures and images for: Development of an openEHR Template for COVID-19 Based on Clinical Guidelines
Source: J Med Internet Res. 2020 Jun 10;22(6):e20239. doi: 10.2196/20239 (PMC7288685; doi:10.2196/20239)

COVID-19 Pneumonia  
Diagnosis and  
Treatment (7th edition)

COMPOSITION: ENCOUNTER

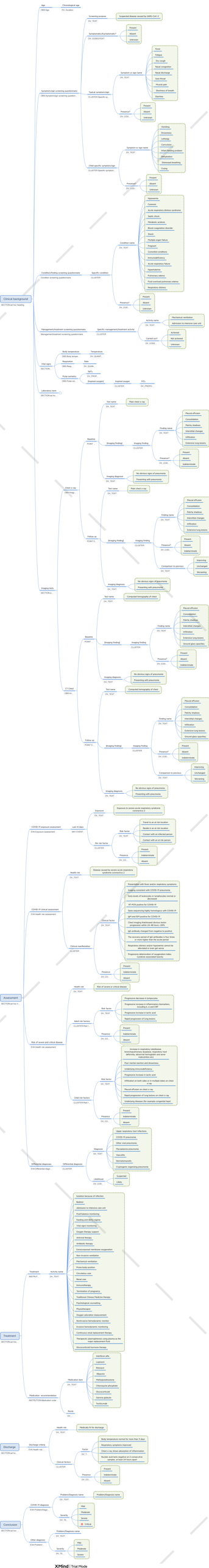

Supplement: Multimedia Appendix 2 [file jmir_v22i6e20239_app2.pdf]
